# Reliability and Validity of a Chinese Version of the Cohen–Mansfield Agitation Inventory-Short Form in Assessing Agitated Behavior

**DOI:** 10.3390/ijerph19159410

**Published:** 2022-08-01

**Authors:** Feng-Ching Sun, Li-Chan Lin, Shu-Chen Chang, Hui-Chi Li, Chia-Hsin Cheng, Ling-Ya Huang

**Affiliations:** 1 Department of Nursing, Kaohsiung Municipal United Hospital, Kaohsiung 80457, Taiwan; sfcmail333@yahoo.com (F.-C.S.); hyy14648@gmail.com (L.-Y.H.); 2 College of Nursing, Fooyin University, Kaohsiung 83102, Taiwan; 3 College of Nursing, Asia University, Taichung 41354, Taiwan; lichan2009@gmail.com; 4 Department of Nursing, Changhua Christian Hospital, Changhua 50006, Taiwan; 53098@cch.org.tw; 5 College of Nursing and Health Sciences, Da-Yeh University, Changhua 515006, Taiwan; 6 Department of Nursing, I-Shou University, Kaohsiung 82445, Taiwan; chbelinda@isu.edu.tw

**Keywords:** agitated behaviors, Cohen–Mansfield Agitation Inventory, dementia, reliability, validity

## Abstract

Background: Patients with dementia often present agitated behaviors. The Cohen–Mansfield Agitation Inventory-short form (CMAI-SF) is one of the most widely used instruments to evaluate agitated behaviors that affect patients’ quality of life and impose burden on caregivers. However, there is no simplified Chinese version of the CMAI-SF (C-CMAI-SF) in clinical settings. Purpose: This study aimed to develop a Chinese version of the C-CMAI-SF and examine its validity and reliability. Methods: This cross-sectional study included three phases. In Phase I, the original CMAI-SF was translated to Chinese. In Phase II, experts were invited to examine the content validity index (CVI). Phase III was conducted to test the validity and reliability of the C-CMAI-SF. Results: The scale showed good validity and reliability with a scale-level CVI of 0.89, Cronbach’s alpha (measure of internal consistency) of 0.874, and test–retest correlation coefficient of 0.902 (for 257 individuals). Using factor analysis, three factors were identified. Regarding concurrent validity, the C-CMAI-SF score was correlated with the Neuropsychiatric Inventory (agitation aggression subscale) and the Cornell Scale for Depression in Dementia (agitation subscale). Conclusions: The study demonstrated that the C-CMAI-SF is a valid and reliable instrument for evaluating agitated behaviors in people with dementia. Relevance to clinical practice: The C-CMAI-SF is an easy and quick tool used to identify and evaluate agitated behaviors in busy clinical settings.

## 1. Introduction

Prior studies show that around 6.97% of people aged 50 and above suffer from dementia [1]. In Taiwan, the percentage of elderly suffering from dementia is around 6% [2]. Nearly 55 million people live with dementia and 10 million new cases are reported every year [3]. This population is expected to reach 78 million in 2030 and 139 million in 2050 [3]. The neuro-anatomical or neuro chemical abnormalities of people with dementia usually result in agitated behaviors [4]. Around 76% of patients with Alzheimer’s disease demonstrate agitated behaviors [5].

Agitated behavior is defined as “inappropriate verbal, vocal, or motor activity that is not judged by an outside observer to result directly from the needs or confusion of the agitated individual” [6]. These behaviors include restlessness, pacing, arguing, disruptive vocalization, and rejection of care [7]. Terms such as aggressive, disturbing, problematic, disruptive behaviors, and restlessness have been used in other research on this topic [8,9]. Older age, functional impairment [10], and declining cognitive function influence and aggravate agitated behaviors [11,12].

Caregiver burden, stress, and depression are closely related with agitated behavior [13,14]. Taking care of dementia patients with agitated behaviors, as compared with those without agitated behavior, gives rise to much greater pressure, depression, and behavior upset [15]. Furthermore, caregivers show higher burnout when facing agitated behaviors than facing other symptoms of dementia such as anxiety and delusions [16].

To measure agitated behaviors, instruments such as the Neuropsychiatric Inventory (NPI) [17] and Cornell Scale for Depression in Dementia (CSDD) [18] were used. The NPI has been demonstrated to be a reliable and valid measurement in assessing psychological symptoms of dementia (BPSD) [19]. However, only one agitated-behavior related item is illustrated in the scales such as agitation/aggression in NPI (NPI/AA). Similarly, the CSDD is a reliable and valid instrument to assess depression among patients with dementia [20] with one agitated-behaviors related item: agitation in CSDD (CSDD/A).

The Cohen–Mansfield Agitation Inventory (CMAI) was developed to assess the frequency of manifestations of agitated behaviors [6] and is used in many studies [21]. The original CMAI, consisting of 29 items, has been translated to different languages. In order to increase the convenience of evaluation and reduce the incompleteness of data collection, the CMAI-short form (CMAI-SF) was developed with 14 items and is widely used in research [22,23]. While the CMAI-long form with 29 items has been translated to Chinese language (C-CMAI-LF) [24] and used in clinical settings and research [11,25], the CMAI-SF has not yet been translated to Chinese and validated. To accommodate the hectic clinical settings, this study aims to develop a Chinese version of the CMAI-SF (C-CMAI-SF) as an easy and quick tool to identify and evaluate agitated behavior. Furthermore, the validity and reliability of such tool is assessed.

## 2. Materials and Methods

The development of the C-CMAI-SF was done in three phases. In Phase I, the original CMAI-SF was translated to Chinese (C-CMAI-SF) based on C-CMAI-LF. In Phase II, content validity was assessed by a panel consisting of experts in geriatrics and dementia. In Phase III, the validity and reliability of the C-CMAI-SF was tested.

### 2.1. Phase I: Translation

In Phase I, the original English version of CMAI-SF was translated to Chinese based on the C-CMAI-LF. The latter was developed based on agitated behaviors observed in the Taiwanese culture [24]. Therefore, the first stage included comparing three scales item-wise: the original CMAI-LF, the original CMAI-SF, and the C-CMAI-LF. The common items in CMAI-SF and CMAI-LF were translated following the C-CMAI-LF, while the items which were either not listed or different from the C-CMAI-LF underwent an English to Chinese translation along with instructions and detailed descriptions of behaviors in the CMAI. As this involves terminology of dementia, two dementia nursing experts with good command of English were asked to check the differences between the three scales and performed the translation. Only few terms were translated, such as aggressive behaviors or self-abuse, chewing, tapping, strange movements, and refusal to follow directions. Wording of some terms in Chinese relating to scratching, aggressive spitting, sexual advance, trying to move to a different place (e.g., out of the room, building), repetitive sentences, calls or words, and hoarding things were slightly modified for greater clarity to be more understandable for users. After the translation, all items of the C-CMAI-SF were evaluated by the content validity index (CVI) in Phase II.

### 2.2. Phase II: Content Validity

The CVI can be divided into the item-level CVI (I-CVI) and scale-level CVI (S-CVI). A group of experts from gerontological panels in different centers, consisting of a clinical psychiatric physician, a nurse working with dementia patients, and the chief executive officer of a dementia daycare center, were approached. They were asked to categorize each item (1–4) into two parts: (a) relevance to measurement’s aim and (b) understandability/clarity. To calculate the I-CVI, the number of experts providing a score of 3 or 4 was divided by the total number of experts. The S-CVI was calculated as the average of all the I-CVIs.

### 2.3. Phase III: Test Validity and Reliability of C-CMAI-SF

Reliability was verified by internal consistency and test–retest reliability. Internal consistency was estimated by Cronbach’s alpha. Test–retest reliability was performed twice with a two-week interval. To measure validity, exploratory factor analysis and concurrent validity were used. The questionnaires on NPI and CSDD were included to assess concurrent validity. Items of the NPI/AA and CSDD/A were used for analysis.

#### 2.3.1. Sample and Participants

Cross-sectional data were collected between November 2020 and January 2021. A sample was selected from 12 community daycare centers providing service on weekdays for older people and those with dementia. A daycare nurse helped to screen and assess participants with a (1) Mini-Mental State Exam (MMSE) score of less than 24 and (2) diagnosed with dementia or probable dementia. To avoid inconsistence, the daycare nurse was trained before assessing each behavior.

#### 2.3.2. Instruments

The C-CMAI-SF consisted of 14 items related to agitated behaviors observed over a period of two weeks, which was same as the original CMAI-SF [26]. Each item was rated on a five-point scale as follows: never (=1), less than once a week (=2), once or several times a week (=3), once or several times a day (=4), a few times an hour or continuously for half an hour or more (=5). The higher the scores, the more frequently the agitated behaviors were observed. 

The Chinese version of NPI is a valid measurement to assess behavioral disturbances of people with dementia [19]. It investigates 12 behavioral domains in terms of frequency (score 1–4), severity (score 1–3), and frequency × severity. Higher scores indicate higher disturbed behaviors. One of the behavioral domains, i.e., NPI/AA, was used for concurrent validity analyses.

The CSDD, developed by Alexopoulos, Abrams, Young, and Shamoian [18], is an instrument to assess depression among patients with dementia. It has been translated to Chinese language, with concurrent validity with the Geriatric Depression Scale-short form of 0.32 (*p* < 0.32) and an interrater reliability of Kappa = 0.43–0.89 [20]. The Chinese version of the CSDD comprises 19 items with each item scored between 0–2 (0 = absent, 1 = mild or intermittent, 2 = severe). The item of agitation (CSDD/A) was used for criterion-related validity analysis. 

Cognitive functioning was assessed using the MMSE [27], which comprises 11 questions within the domains of orientation, registration, recall, attention, calculation, understanding and use of language, and praxis. The test has a maximum score of 30 points, with a higher score indicating better cognitive function. Cronbach’s alpha value for this study was 0.86. 

The Barthel Index (BI) was used to measure participants’ performance of activities of daily living (ADL) [28]. It comprises 10 items for assessing self-care ability, with a score range of 0–100. The score 100 indicates fully independent, 91–99—mildly dependent, 61–90—moderately dependent, 21–60—heavily dependent, and 0–20—totally dependent [29].

### 2.4. Statistical Analysis

Statistical Package for the Social Sciences version 20.0 was used to perform the statistical analyses. Cronbach’s alpha coefficient was used to measure the internal consistency of the C-CMAI-SF; the value of Cronbach’s alpha should reach 0.8 [30]. The corrected item-total correlation should be above 0.3 [31]. The test–retest reliability was performed by calculating Pearson’s correlation coefficient.

Factor analysis was used to verify construct validation. This study also calculated the correlations between the C-CMAI-SF sum score and each item of the C-CMAI-SF with NPI/AA and CSDD/A, using Pearson’s correlation coefficient to determine concurrent validity.

## 3. Results

### 3.1. Participant Characteristics

The study was conducted with 257 people aged 57–102 years, with a mean age of 79.97 (±8.81) years. More than half of the participants were women (62.26%). The majority of the participants were primary school-educated. The mean values (± standard deviation (SD)) of MMSE and ADLs were 10.28 (± 7.38) and 69.22 (±24.51), respectively, (Table 1).

Around 71.60% of the participants demonstrated any one agitated behaviors at least once a week. The mean number of items was 1.40, with SD of 0.53. The percentage of the participants with agitated behaviors at least once a week was also calculated. The top three most frequently demonstrated agitated behaviors in decreasing order were Item 5—pace, aimless wandering, trying to move to a different place (43.58%), Item 6—general restlessness, performing repetitious mannerisms, tapping, strange movements (33.07%), and Item 1—cursing or verbal aggression (29.18%) (Table 2).

### 3.2. Phase II: Content Validity

The CVI of the measurement items was computed to assess content validity. The I-CVI of each item in the “relevance to measurement’s aim” part was 1.0, and that in the “understandability/clarity” part ranged from −0.67 to 1.0. The S-CVI was 0.79 for 14 items in the “understandability/clarity” part. These reflect good content validity. 

### 3.3. Phase III: Testing Reliability and Validity of C-CMAI-SF

#### 3.3.1. Internal Consistency

Cronbach’s alpha value for total CMAI was 0.874. After omitting some items from the CMAI, Cronbach’s alpha ranged from 0.852 to 0.877, indicating high reliability. The corrected item-total correlation was above 0.3 (Table 3).

#### 3.3.2. Test–Retest Reliability

Test–retest reliability was computed by Pearson’s correlation coefficient of the total CMAI scores. The correlation coefficient between the initial score and the retest score with a two-week interval was 0.902, indicating good correlation.

#### 3.3.3. Construct Validity 

Factor analysis was carried out using a principal axis with varimax rotation. Fourteen items were included in this category. Rotation sums of squared loadings explain 61.08% of the total variance. The Kaiser–Meyer–Olkin (KMO) measure of sampling adequacy was 0.87, and Bartlett’s sphericity test was 1599.674 (*p* < 0.01). The KMO value exceeded the suggested value of 0.6 [32,33], and Bartlett’s sphericity test reached statistical significance [34]. indicated suitability for performing factor analysis. The result identified three factors—Factor 1: verbally agitated behaviors; Factor 2: aggressive behaviors; and Factor 3: physically non-aggressive behaviors—as suggested by [26,35] (Table 4).

#### 3.3.4. Concurrent Validity

Pearson’s correlation coefficient was used to compare the total CMAI score and scores of each item to the NPI/AA and CSDD/A values. Results showed that there was a significant correlation between CMAI sum and total score of NPI/AA frequency (*r* = 0.575, *p* < 0.01), NPI/AA intensity (*r* = 0.481, *p* < 0.01), NPI/AA frequency × intensity (*r* = 0.203, *p* < 0.01), and CSDD/A (*r* = 0.561, *p* < 0.01). There was no significant correlation between items corresponding to CMAI 5, CMAI 7, CMAI 8, CMAI 10, CMAI 12, CMAI 13, and CMAI 14 with the total score of NPI/AA frequency × intensity (Table 5).

## 4. Discussion

With an increasing number of people suffering from dementia and agitated behaviors, a reliable and convenient instrument is required for health caregivers. The aim of this study was to translate the original English version of the CMAI-SF to Chinese based on the C-CMAI-LF. The current study developed a 14-item C-CMAI-SF for testing agitated behaviors in clinical settings. The results show that the C-CMAI-SF has high reliability and validity by using different statistical methods. The reliability showed adequate results by analyzing internal consistency, corrected item-total correlation, and test–retest reliability. The total internal consistency score was 0.874 and corrected item-total correlation was above 0.3. The correlation coefficient or test–retest was 0.902. The validity of C-CMAI-SF is supported by good content validity (I-CVI: 0.83–1.0; S-CVI: 0.89), construct validity, and concurrent validity. Construct validity was based on factor analysis wherein three factors were identified, and was consistent with the original version. The concurrent validity correlated with two agitated indices—NPI/AA and CSDD/A—revealed moderate validity.

The current study showed that the prevalence of agitated behavior was 71.60% among people with dementia. The most frequently demonstrated agitated behaviors were pace, aimless wandering, trying to move to a different place, general restlessness, performing repetitious mannerisms, tapping, strange movements, and cursing or verbal aggression. This result is similar to that of a study by Veldwijk-Rouwenhorst, et al. [36] indicating that these could be the most common agitated behaviors among people with dementia.

Internal consistency was measured by Cronbach’s alpha. A Cronbach’s alpha of 0.80 or higher is acceptable for a general instrument [30]. In this study, Cronbach’s alpha for the total CMAI was 0.874. If a certain item is deleted, its internal consistency is higher than the sum of internal consistency, indicating that the internal consistency of this item is poorer than that of other items [37]. When Item 5 of the C-CMAI-SF was deleted in the current study, Cronbach’s alpha was 0.877, which is higher than the sum of internal consistency. However, if the original internal consistency reached the optimum level and the internal consistency of the deleted item does not differ from the original and is greater than 0.80, then it is not necessary to delete that item [37]. Therefore, Item 5 was retained in the instrument. Moreover, the corrected item-total correlation was above 0.3, which means that the subset of 14 items was highly correlated with the full form of the C-CMAI-SF [38]. Test–retest reliability was used to confirm that the instrument was stable over time. The correlation coefficient of test–retest in this study was 0.902 over a two-week interval, which exceeds 0.6 and indicates good correlation [39]. 

The content validity of C-CMAI-SF was confirmed by the CVI value and was carefully examined by an expert panel. Experts including a doctor specialized in dementia, a nursing home nurse, and a chief executive officer of a daycare center were invited. Therefore, the content validity of C-CMAI-SF was confirmed by diverse dementia specialists. Moreover, an I-CVI higher than 0.78 for three or more experts can be considered as good evidence of content validity [40]. Content validity was tested by I-CVI, which ranged from 0.83 to 1.0, and S-CVI was 0.89, which is considered good content validity. 

Factor analysis was conducted to identify the underlying dimensions of the instrument. These dimensions were represented as theoretical constructs [41]. The CMAI-SF was developed based on the original CMAI-LF, which was developed according to one-to-one nursing staff interviews and rating of each nursing home resident [6]. The C-CMAI-SF in the current study reflects three core dimensions which are consistent with the original study and the previous study. The original CMAI comprises three core dimensions [26]. The instrument development of the CMAI-SF for people with impaired cognitive function in nursing homes also identified three factor dimensions [42]. Furthermore, the CMAI-LF among people with dementia in Hong Kong also consists of three dimensions [35]. 

Factor loadings are the regression weights or predictors of the measured variables from these dimensions. The stability of factor solutions was determined by the size of the factor loadings, along with the total sample size and the number of indicators per factor [41]. In this study, factor loading was between 0.53 and 0.82 for a sample size of 257, which indicates that solutions were highly stable across replicated samples when each component contained at least 14 variables. This result is similar to the C-CMAI-LF used for elderly people in Hong Kong [35], which showed a factor loading between 0.346 and 0.785. 

Although the results of factor analysis of the current study led to the extraction of three factors, which is consistent with the original CMAI, some items can be classified as having different dimensions compared to other studies. Item 14 in this study (screaming) can be categorized as an aggressive behavior, which is inconsistent with “verbal agitated behaviors” as listed in the original CMAI [26]. However, the result of the current study is similar to the C-CMAI-LF administered in Hong Kong, which categorizes “screaming” as “physical aggressive behaviors” [35]. A possible reason is cultural difference. Screaming is not only seen as an incorrect language behavior, but also as an abnormal reaction. Therefore, “screaming” could be categorized as aggressive behavior instead of “verbal aggressive” in our study. Item 6 (general restlessness, performing repetitious mannerism, tapping, strange movements) in the current study was grouped as verbal agitation, but this item belonged to the “physically non-aggressive behavior” group in the original CMAI [26]. The possible reason was people with dementia express their demands or requests by showing restlessness, tapping, and strange movements, which were therefore classified as “verbal aggressive.” In a similar study based in Hong Kong [35], general restlessness was listed under “physically non-aggressive behavior”, with factor loading of 0.434, and was also grouped under “verbally agitated behavior”, with a factor loading of 0.383.

The concurrent validity of the C-CMAI-SF was verified in this study. The comparison of the NPI/AA and CSDD/A was supported by a previous study [21]. This study found that total sum of the C-CMAI-SF was significantly correlated with the NPI/AA and CSDD/A. The results of the current study were similar to those of [21]. However, when individual items of the C-CMAI-SF were compared to both the instruments, the CSDD/A showed better correlation with NPI/AA frequency × intensity. The individual items of the C-CMAI-SF (5,7,8,10,12,13,14) did not have significant correlation with NPI/AA frequency × intensity. A possible reason is that certain items of the C-CMAI-SF (5,7,8,13) belong to core dimensions of physically non-aggressive behavior, which may not be exactly the same as to NPI aggression behaviors. For similar reasons, items 10 (repetitive sentences, calls or words), 12 (strange noise), and 14 (screaming) may indicate verbal disorders instead of aggressive or agitated behaviors. When different scales are used, comparison becomes difficult [21].

### Limitation

This study encompasses certain limitations which need to be addressed. First, the participants were selected from daycare centers in southern Taiwan and may not represent all types of agitated behaviors demonstrated by people with dementia. The scope of generalization of the findings of this study in other areas may be restricted, as categorization of each item in different core dimensions varied with the culture in question. Second, the presence of agitated behaviors is closely connected to cognitive level; therefore, the results may vary depending on individuals’ cognitive functioning. Owing to the limited sample size, this aspect could not be analyzed fully. Further studies need to explore the reliability and validity of this instrument after taking into consideration various cognitive functions. Although the daycare nurse was trained before assessing each behavior, inconsistencies might still occur, especially considering that the dementia case could be difficult to articulate. This suggests that a standardization test should be performed for further studies to improve uniform data collection.

## 5. Conclusions

People with dementia usually have a high prevalence of agitated behaviors. The study demonstrated that the C-CMAI-SF is a valid and reliable instrument for evaluating agitated behaviors. The C-CMAI-SF is an instrument offering an easy and quick assessment in clinical settings. Cultural differences should be considered in the development of this instrument, especially when factor analysis is used.

## Figures and Tables

**Table 1:** Demographic features of the study subjects.

Items	Number	%
Gender		
Male	97	37.74%
Female	160	62.26%
Educational Status		
Illiterate	80	31.13%
Primary	103	40.08%
High School	61	23.74%
College/Higher	13	5.06%
	Mean ± SD	Range
Age (Years)	79.97 ± 8.81	57–102
MMSE	10.28 ± 7.38	0–24
ADLs	69.22 ± 24.51	0–100

**Table 2:** The frequency and mean score in individual items of CMAI.

	%	Mean	SD
1. Cursing or verbal aggression	29.18	1.47	0.84
2. Hitting (including self), kicking, pushing, biting, scratching, aggressive, spitting (include at meals)	16.34	1.30	0.77
3. Grabbing onto people, throwing things, tearing things or destroying property	12.84	1.23	0.67
4. Other aggressive behavior or self abuse including: intentional falling, making verbal or physical sexual advances, eating/drinking/chewing inappropriate substances, hurting self or others	8.17	1.12	0.46
5. Pace, aimless wandering, trying to move to a different place (e.g., out of the room, building)	43.58	2.03	1.36
6. General restlessness, performing repetitious mannerisms, tapping, strange movements	33.07	1.65	1.08
7.Inappropriate dress or disrobing	11.28	1.22	0.71
8. Handling things inappropriately	21.01	1.41	0.88
9. Constant request for attention or help	21.01	1.51	0.96
10. Repetitive sentences, calls or words	26.46	1.60	1.10
11. Complaining, negativism, refusal to follow direction	24.90	1.47	0.88
12. Strange noises, (weird laughter or crying)	11.28	1.21	0.66
13. Hiding things, hoarding things	18.29	1.38	0.90
14. Screaming	3.89	1.06	0.34

**Table 3:** Internal reliability for C-CMAI-SF analysis.

	Corrected Item-Total Correlation	Cronbach’s Alpha if Item Deleted
CMAI 1	0.652	0.863
CMAI 2	0.597	0.866
CMAI 3	0.739	0.860
CMAI 4	0.459	0.872
CMAI 5	0.590	0.877
CMAI 6	0.808	0.852
CMAI 7	0.505	0.870
CMAI 8	0.673	0.862
CMAI 9	0.747	0.857
CMAI 10	0.673	0.864
CMAI 11	0.697	0.860
CMAI 12	0.596	0.866
CMAI 13	0.557	0.869
CMAI 14	0.475	0.872
Total Cronbach’s alpha = 0.874		

**Table 4:** Factor structure of the C-CMAI-SF using exploratory factor analysis.

	Factor 1	Factor 2	Factor 3
CMAI 10	0.820		
CMAI 9	0.818		
CMAI 6	0.758		0.367
CMAI 11	0.637		
CMAI 4		0.741	
CMAI 14	0.353	0.696	
CMAI 3		0.610	0.530
CMAI 2		0.589	
CMAI 12	0.496	0.564	
CMAI 1	0.472	0.531	
CMAI 8			0.760
CMAI 5	0.339		0.711
CMAI 13			0.698
CMAI 7			0.614
Explained variance	22.9%	19.14%	19.06%
Total explained variance			61.08%

**Table 5:** Concurrent correlation of the CMAI with the NPI/AA and CSDD/A.

	NPI/AA Frequency	NPI/AA Intensity	NPI/AA Frequency × Intensity	CSDD/Agitation
CMAI sum	0.575 **	0.481 **	0.203 **	0.561 **
CMAI 1	0.628 **	0.560 **	0.332 **	0.354 **
CMAI 2	0.412 **	0.355 **	0.134 *	0.316 **
CMAI 3	0.420 **	0.335 **	0.143 *	0.397 **
CMAI 4	0.409 **	0.332 **	0.162 **	0.220 **
CMAI 5	0.250 **	0.240 **	0.067	0.421 **
CMAI 6	0.463 **	0.374 **	0.144 *	0.497 **
CMAI 7	0.178 **	0.170 **	0.049	0.377 **
CMAI 8	0.297 **	0.257 **	0.082	0.420 **
CMAI 9	0.408 **	0.338 **	0.124 *	0.373 **
CMAI 10	0.308 **	0.223 **	0.079	0.358 **
CMAI 11	0.478 **	0.413 **	0.250 **	0.376 **
CMAI 12	0.401 **	0.269 **	0.121	0.323 **
CMAI 13	0.241 **	0.238 **	0.077	0.211 **
CMAI 14	0.345 **	0.201 **	0.109	0.140 *

* *p* < 0.05, ** *p* < 0.01.

## Data Availability

The data presented in this study are available on request from the corresponding author.
